# Predictors of New-Onset Epilepsy in People With Younger-Onset Neurocognitive Disorders

**DOI:** 10.3389/fnagi.2021.637260

**Published:** 2021-03-16

**Authors:** Xinshi Wang, Samantha M. Loi, Emma Foster, Zhibin Chen, Dennis Velakoulis, Patrick Kwan

**Affiliations:** ^1^Department of Neurology, The First Affiliated Hospital of Wenzhou Medical University, Wenzhou, China; ^2^Department of Neuroscience, The Central Clinical School, Monash University, Melbourne, VIC, Australia; ^3^Department of Neurology, The Royal Melbourne Hospital, The University of Melbourne, Melbourne, VIC, Australia; ^4^Neuropsychiatry, The Royal Melbourne Hospital and Melbourne Neuropsychiatry Center, The University of Melbourne and The Royal Melbourne Hospital, Melbourne, VIC, Australia; ^5^School of Public Health and Preventive Medicine, Monash University, Melbourne, VIC, Australia

**Keywords:** epilepsy, predictors, neurocognitive disorders, prevalence study, case control study, young-onset

## Abstract

**Objective:** People with neurocognitive disorders (NCDs) have an increased risk of epilepsy. However, most studies investigating the risk of seizures in people with NCDs are limited to those with Alzheimer's disease (AD) and vascular dementia (VD), and those who developed dementia after age 65 years. A knowledge gap exists regarding factors associated with development of epilepsy in people with younger-onset NCD, and those with non-AD and non-VD dementia subtypes. In this study, we aimed to identify the factors associated with the development of epilepsy in people with younger-onset NCDs of varied etiologies, the majority of whom had symptom onset prior to age 65 years.

**Participants and Methods:** This was a retrospective study reviewing the medical records of consecutive people admitted with cognitive impairment to a tertiary neuropsychiatry unit between 1 January 2004 and 30 April 2019. People diagnosed with primary NCDs were included in the analysis. The prevalence and characteristics of epilepsy were described. The factors associated with developing epilepsy were identified in a binary logistic regression model.

**Results:** A total of 427 people were included. One hundred fourteen had Alzheimer's disease, 104 frontotemporal dementia, 51 vascular dementia, 69 movement disorder-associated dementia, and 89 unspecified NCD. The median age on admission was 59 years (range 33–86) and 75.2% (*n* = 321/427) had young-onset NCD with onset before 65 years of age. 40/427 (9.4%) people had epilepsy, and epilepsy onset clustered between 2 years before and 6 years after the onset of cognitive decline in 80% (*n* = 32/40). The most frequent seizure type was focal to bilateral tonic-clonic seizure (35%, *n* = 14/40). Most of the people (94.7%, *n* = 36/38) achieved seizure freedom with one or two antiseizure medications. People with unspecified NCD (compared to frontotemporal dementia and movement disorder-associated dementia, age of onset of NCDs ≤50 years, and current smoking status were independently associated with higher risk of developing epilepsy.

**Conclusion:** Epilepsy is common in people with younger-onset NCDs, and a high index of suspicion is warranted particularly for those with unspecified subtype and smoking status. Smoking reduction or cessation should be further investigated as a potentially modifiable factor for risk reduction.

## Introduction

Neurocognitive disorders (NCDs) are characterized by substantial decline in one or more cognitive domains including complex attention, executive function, learning and memory, language, perceptual-motor, and social cognition, not attributable to delirium or other mental disorders (American Psychiatric Association, 2013). Studies have shown that people with NCDs have an increased risk of seizures, in the order of 6.2–10-fold for Alzheimer's disease (AD) (Hesdorffer et al., 1996; Friedman et al., 2012; Imfeld et al., 2013; Vossel et al., 2017), 5.7-fold for vascular dementia (VD) (Imfeld et al., 2013) and 8-fold for non-AD dementia (Hesdorffer et al., 1996). Furthermore, people with NCD and epilepsy present with cognitive impairment earlier than those with NCD alone (Vossel et al., 2013). Several studies have demonstrated that decline in cognitive function is faster in those with seizures compared to those without seizures (Volicer et al., 1995; Lott et al., 2012). These findings underscore the importance of rapid diagnosis and treatment of new-onset epilepsy, with the view to achieve seizure control early, in people with NCDs.

There are a small number of studies exploring potential risk factors for the development of epilepsy in people with NCDs. Some of these risk factors include hyperlipidemia (Bernardi et al., 2010), male sex (Bernardi et al., 2010), longer duration of dementia for AD (Imfeld et al., 2013), shorter duration of dementia for VD (Imfeld et al., 2013), more severe cognitive impairment (McAreavey et al., 1992), and younger age at onset of cognitive decline or point of dementia diagnosis (McAreavey et al., 1992; Amatniek et al., 2006; Vossel et al., 2013; Beagle et al., 2017; Zelano et al., 2020). Furthermore, many recent studies find that seizures cluster at the early stage of cognitive impairment, or precede the onset of cognitive decline (Vossel et al., 2013; Beagle et al., 2017; Zelano et al., 2020).

However, most studies investigating factors associated with seizures in people with NCDs are limited to cohorts with AD and VD (Hesdorffer et al., 1996; Friedman et al., 2012; Imfeld et al., 2013; Vossel et al., 2017). Although younger age at onset of cognitive decline had been the most commonly identified risk factor for developing epilepsy in those with NCDs (McAreavey et al., 1992; Amatniek et al., 2006; Vossel et al., 2013; Beagle et al., 2017; Zelano et al., 2020), these study cohorts include participants with a mean age that is still more than 65 years of age. Thus, a knowledge gap exists regarding factors associated with the development of epilepsy in people with younger-onset NCDs, and those with non-AD and non-VD NCD subtypes.

In this study, we aimed to identify the factors associated with the development of epilepsy in people with younger-onset NCDs. We included a cohort of people with NCDs of varied etiologies, the majority of whom had symptom onset prior to age 65 years.

## Participants and Methods

### Subjects

This retrospective study was based on medical record review of consecutive people with cognitive decline who attended The Royal Melbourne Hospital (RMH) Neuropsychiatry Unit, Melbourne, Australia between 1 January 2004 and 30 April 2019. This Neuropsychiatry Unit is a state-based tertiary referral center with a special interest in young-onset NCDs. It comprises an eight-bed diagnostic inpatient unit as well as outpatient specialist clinics.

Primary inclusion criterion for the study were people who fulfilled the Diagnostic and Statistical Manual of Mental Disorders (DSM)-5 NCD diagnostic criteria (American Psychiatric Association, 2013). Those with likely NCD-related epilepsy were identified on review of medical records by consensus agreement of two epileptologists (X.W. and E.F.). Disagreements were adjudicated by a panel of senior clinicians: epileptologist (P.K.) and neuropsychiatrists (S.L. and D.V.). We excluded people whose cognitive impairment and/or epilepsy was due to established acquired brain injuries (ABIs) such as stroke, tumor, and traumatic brain injury; people diagnosed with specific NCDs with known genetic causes (e.g., Huntington's disease, Niemann-Pick type C disease, and Down syndrome-associated AD), as these NCDs were considered to be due to unique biological mechanisms and symptoms differed from the sporadic forms of young-onset NCDs. Individuals with familial forms of AD, e.g., conferred by presence of APOE ε4 allele, were not excluded, as the underlying pathology was considered similar to sporadic NCD cases. We also excluded people whose cognitive impairment was attributed to uncontrolled epilepsy and/or ASMs, rather than their NCDs, and those whose epilepsy occurred more than 10 years before the onset of cognitive impairment (Vossel et al., 2013). The study was approved by The RMH Human Research Ethics Committee (QA2012044).

### Variables Collected

#### NCD Variables

NCDs were categorized as “mild” or “major” depending on whether functional independence was impaired, with mild NCD corresponding to mild cognitive impairment (MCI) and major NCD corresponding to dementia (American Psychiatric Association, 2013). Where possible, cases were further categorized into specific etiologic subtypes, based on international guidelines and diagnostic criteria. These included AD (McKhann et al., 1984); frontotemporal dementia (FTD), including behavior variant FTD (bvFTD) (Rascovsky et al., 2011) and primary progressive aphasia (PPA) (Gorno-Tempini et al., 2011) subtypes; VD (Roman et al., 1993); dementia with Lewy bodies (DLB) (McKeith et al., 1996); Parkinson's disease dementia (PDD) (Emre et al., 2007); dementia associated with multiple system atrophy (MSA) (Kitayama et al., 2009); dementia associated with progressive supranuclear palsy (PSP) (Golbe, 2014); dementia associated with corticobasal degeneration (CBD) (Dickson et al., 2002); and unspecified NCD which included mixed dementias and dementias unable to be ascribed to a specific subtype. The age at onset of cognitive decline was obtained from the study participants or their caregivers.

Cognitive function was assessed using the Neuropsychiatry Unit Cognitive Assessment Tool (NUCOG), which is a validated screening tool testing five cognitive domains: attention, visuoconstructional function, memory, executive function, and language. Each cognitive domain is scored out of 20, producing a total score between 0 and 100, with higher scores indicating better cognitive function. It has been demonstrated that a cut-score of 80 is more sensitive and specific for the detection of dementia and differentiation of dementia subgroups than the Mini-Mental State Examination (MMSE) (Walterfang et al., 2006). For each patient, the NUCOG score obtained at the last follow-up (for people without seizures) or on the date nearest to seizure onset was included in the analysis.

Duration of cognitive decline was defined as the period between the onset date of cognitive decline and the date of last follow-up (for people without seizures) or the date of follow-up nearest seizure onset. Structural neuroimaging findings based on head magnetic resonance imaging (MRI) and computer tomography (CT) scans with dementia-protocols were classified as either “normal,” “demonstrating cortical atrophy,” or “demonstrating non-specific abnormalities” such as small-vessel ischemic change. Functional neuroimaging findings based on single-photon emission computed tomography (SPECT) were defined as abnormal if demonstrating hypoperfusion in any brain region. Prescription of anticholinesterase inhibitors, such as donepezil, rivastigmine, and memantine, was recorded.

#### Other Variables That Might Be Potential Risk Factors for Epilepsy

Other variables collected included: (1) demographic information, including age, sex, level of educational attainment, and marital status; (2) medical and psychiatric history, with specific note of hypertension, diabetes, hypercholesterolemia, obstructive sleep apnea, depression/anxiety and “other” psychiatric disorders (e.g., schizophrenia); and (3) lifestyle factors including current smoking status (defined as smoking within 12 months prior to recognition of cognitive decline) and current alcohol use (defined as drinking at least 1 standard unit of alcohol a week) (Topiwala et al., 2017). For people without epilepsy, data were drawn at the time point of the most recent follow-up appointment. For people with epilepsy, data were drawn at the time point just prior to seizure onset.

#### Variables Related to Epilepsy

Epilepsy was diagnosed according to the latest criteria set out by the International League Against Epilepsy (ILAE): two or more unprovoked seizures separated by at least 24 h, or one unprovoked seizure with an increased risk of recurrent seizures (Fisher et al., 2014), as evidenced by the presence of epileptiform activity on electroencephalography (EEG). We conservatively excluded patients with ambiguous events.

The characteristics of epilepsy were recorded, including age at onset of epilepsy, types of seizures, types and number of antiseizure medications (ASMs) prescribed, and seizure control. Types of seizures were classified as per the ILAE classification, and included focal aware seizure (FAS), focal impaired awareness seizure (FIAS), focal to bilateral tonic-clonic seizure (FBTCS), and generalized tonic-clonic seizure (GTCS) (Scheffer et al., 2017). Seizure semiology was also classified as convulsive vs. non-convulsive.

### Statistical Analysis

We performed univariable comparisons of clinical variables between people with and without epilepsy using Fisher exact test, Student *t*-test, or Mann-Whitney *U*-test, as appropriate. Variables with *p* < 0.2 were entered into the multivariable logistic regression and forward stepwise method was used to establish the fitted model. For the purposes of analysis, people with DLB, PDD, MSA, PSP, and CBD were grouped together as movement disorder associated dementia (MDD). Two-tailed *p* < 0.05 were considered statistically significant. All statistical analyses were performed using the software package SPSS version 23.0.

## Results

**Figure 1:**
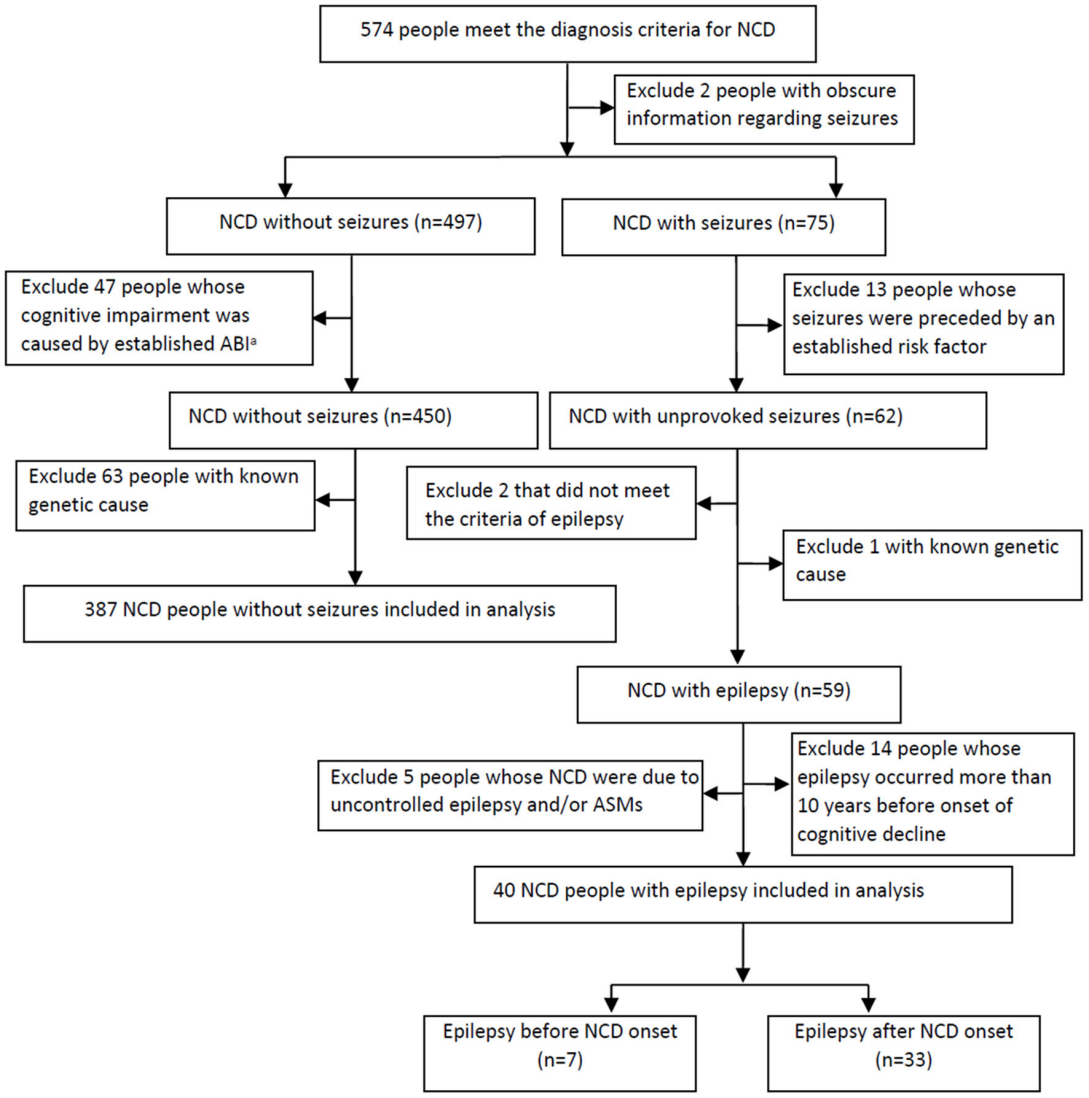
Study flow chart of subject collection. NCD, neurocognitive disorder; ABI, acquired brain injury; ASMs, antiseizure medications.

A total of 575 people met the diagnostic criteria for NCDs. Among them, 427 people fulfilled all the inclusion criteria and were included in analysis. The final cohort was comprised of 67 people (15.7%) with mild NCD and 398 (84.3%) with major NCD. Of these, 40 (9.4%) had epilepsy and 387 (90.6%) did not have epilepsy. Figure 1 displays a flowchart of people included and excluded from the study.

### Characteristics of All People With NCDs

The median age on admission to the Neuropsychiatry inpatient unit was 59 years (range 33–86). The median age of NCD onset was 57 years (range 32–86), and 75.2% (*n* = 321/427) were diagnosed as young-onset NCDs with onset before 65 years of age (Rossor et al., 2010). One hundred fourteen people had AD, 104 FTD, 51 VD, 69 MDD (comprised of 24 DLB, 33 PDD, 3 MSA, 4 PSP, and 5 CBD), and 89 unspecified NCD (including 24 people with mixed dementia, 14 people with mild cognitive impairment who did not meet diagnostic criteria for differential diagnoses, and 51 people who did not meet the criterion for a specific NCD type). The median duration of cognitive decline was 3 years (range 0.5–15 years) prior to inclusion in the study. A total of 324 (75.9%) people had undergone NUCOG assessment and the mean NUCOG score was 66.1 (standard deviation [SD] 17.5). Four hundred twenty-two people (98.8%) underwent neuroimaging, including 412 MRI scans and 10 CT scans, and 368 people (86.2%) had SPECT scans. Detailed characteristics of the whole cohort are displayed in Table 1.

**Table 1:** Baseline characteristics of the whole cohort.

**Variables**	**Characteristics**
**Demographics**	
Female sex, No./total No. (%)	194/427 (45.4)
Age, median (range)	59 (33–86)
**Education, No./total No. (%)**	
No more than basic	90/419 (21.5)
High school	189/419 (45.1)
Tertiary	140/419 (33.4)
Married, No./total No. (%)	285/427 (66.7)
**NCD characteristics**	
Mild NCD, No./total No. (%)	67/427 (15.7)
**Dementia subtypes, No./total No. (%)**	
AD	114/427 (26.7)
VD	51/427 (11.9)
MDD	69/427 (16.2)
DLB	24/427 (5.6)
PDD	33/427 (7.7)
MSA	3/427 (0.7)
PSP	4/427 (0.9)
CBD	5/427 (1.2)
FTD	104/427 (24.4)
Unspecified^a^	89/427 (20.8)
**NCD onset age, No./total No. (%)**	
Young-onset (<65)	321/427 (75.2)
≤50	116/427 (27.2)
NCD Duration (years), median (range)	3.00 (0.5–15)
Total NUCOG^b^, mean ± SD	66.10 ± 17.50
Family history of NCD, No./total No. (%)	95/427 (22.2)
**Neuroimaging**^**c**^**, No./total No. (%)**	
Abnormality	363/422 (86.0)
Cortical atrophy	259/422 (61.4)
Non-specific abnormality	226/422 (53.6)
Small-vessel ischemic change	170/422 (40.3)
SPECT abnormality^d^, No./total No. (%)	333/368 (90.5)
On ADM^e^, No./total No. (%)	62/365 (17.0)
**Medical history**	
**Psychiatric history, No./total No. (%)**	
Depression/anxiety	163/427 (38.2)
Other psychiatric disorders	78/427 (18.3)
Hypertension, No./total No. (%)	125/427 (29.3)
Diabetes, No./total No. (%)	62/427 (14.5)
Hypercholesterolaemia, No./total No. (%)	172/427 (40.3)
Obstructive sleep apnea, No./total No. (%)	24/427 (56.2)
**Lifestyle factors**	
Current smoking, No./total No. (%)	78/427 (18.3)
Alcohol use, No./total No. (%)	109/427 (25.5)

*AD, Alzheimer disease; VD, Vascular dementia; MD, Movement disorder associated dementia; DLB, Dementia with Lewy bodies; PDD, Dementia associated with Parkinson disease; MSA, Multiple system atrophy; PSP, progressive supranuclear palsy; CBD, corticobasal degeneration; FTD, frontotemporal dementia; ADM, anti-dementia medications*.

a *include 24 people with mixed NCDs (18 VD/AD, 3 FTD/VD, 2 PDD/VD, and 1 DLB/VD), 14 people with mild cognitive impairment who did not meet diagnostic criteria for differential diagnoses, and 51 people who did not meet the criterion for a specific type*.

b *data available for 324 people*.

c *Mostly based on MRI, 10 of them based on CT as the individuals (including 2 people with epilepsy) had only CT but no MRI, 5 people (all without epilepsy) had neither MRI nor CT*.

d *data available for 368 people*.

e *before onset of epilepsy or before the last follow-up record*.

### Clinical Characteristics of NCD People With Epilepsy

**Figure 2:**
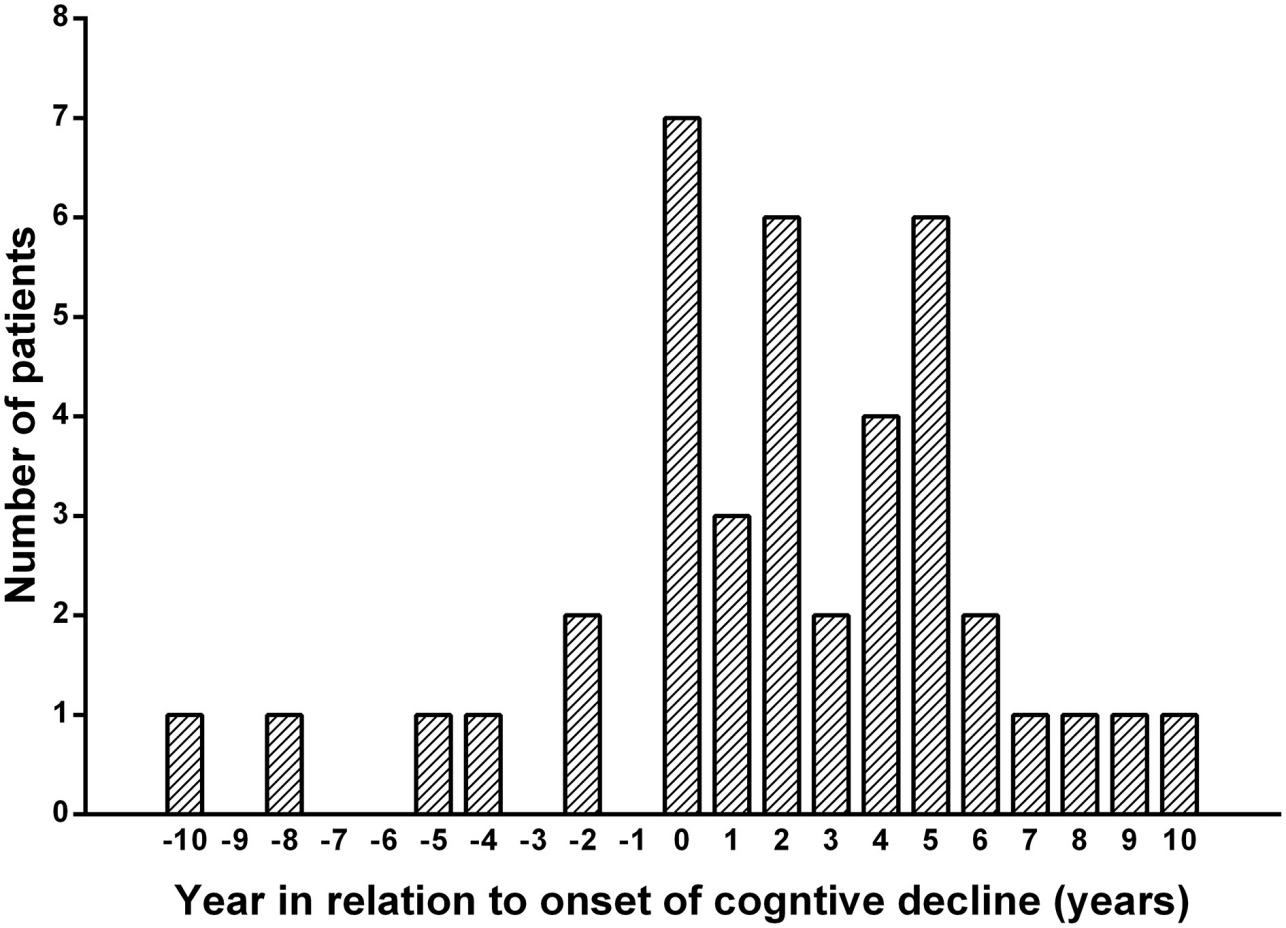
Onset of epilepsy in relation to the onset of cognitive decline. The yearly distribution of new-onset seizures relative to the year of onset of cognitive decline: the median duration between the onset of cognitive decline and the onset of epileptic seizures was 2 years (range −10 to 10 years), with 80% of people (*n* = 32/40) experiencing their first epileptic seizures within the time frame ranging from 2 years prior through to 6 years after the onset of cognitive decline.

Among the study cohort, 40 (9.4%) people had epilepsy. Their characteristics are shown in Table 2. The median age at onset was 57.5 (range 30–87) and 75.2% (*n* = 321/427) of people fulfilled the diagnostic criteria for young-onset NCDs with the age of onset <65 years (Rossor et al., 2010). Seven (17.5%) people developed epilepsy prior to cognitive decline and 33 (82.5%) after cognitive decline. The median duration between the onset of cognitive decline and the onset of epileptic seizures was 2 years (range −10 to 10 years), with 80% of people (*n* = 32/40) experiencing their first epileptic seizures within the time frame ranging from 2 years prior through to 6 years after the onset of cognitive decline (Figure 2).

**Table 2:** Clinical characteristics of people with epilepsy (*n* = 40).

**Variables**	**Characteristics**
Age at onset of epilepsy (years), median (range)	57.5 (30–86)
Female sex, No./total No. (%)	16/40 (40.0)
Epilepsy after NCD onset, No./total No. (%)	33/40 (82.5)
Duration between cognitive decline and onset of epilepsy (years), median (range)	2.00 (−10, 10)
**Seizure type, No./total No. (%)**	
FAS	2/40 (5.0)
FIAS	13/40 (32.5)
FBTCS	14/40^#^ (35.0)
GTCS	11/40 (27.5)
NCS	11/40 (27.5)
**ASM regimens, No./total No. (%)**	
Monotherapy	30/40 (75)
Polytherapy	8/40 (20)
Specific ASMs:	
• VPA	9/40 (22.5)
• LEV	7/40 (17.5)
• PHT	6/40 (15.0)
• CBZ	6/40 (15.0)
• LTG	2/40 (5.0)
• LTG+VPA	3/40 (7.5)
• LTG+PTH	1/40 (2.5)
• LEV+VPA	1/40 (2.5)
• CBZ+VPA	1/40 (2.5)
• CBZ+LEV+PTH+ZNS	1/40 (2.5)
• Clobazam+LEV+PTH+VPA	1/40 (2.5)
No ASM	2/40 (5.0)

*FAS, focal aware seizure; FIAS, focal impaired awareness seizure; NCS, non-convulsive seizure; FBTCS, focal to bilateral tonic-clonic seizure; GTCS, generalized tonic-clonic seizure; VPA, valproate; LEV, levetiracetam; PHT, phenytoin; CBZ, carbamazepine; LTG, lamotrigine; ZNS, Zonisamide; ASM, anti-seizure medication*.

# *nine of them had both FIAS and secondary generalization*.

Regarding the semiology of seizures, 2 (5%) people had FAS only, 13 (32.5%) had FIAS only, 14 (35%) had FBTCS (including 9 with both FIAS and FBTCS, and 5 with FBTCS only), and 11 (27.5%) had GTCS. Eleven of the 13 people (84.6%) with FIAS has non-convulsive seizures without motor symptoms. Among the people who had seizures before dementia onset, most (*n* = 6/7, 85.7%) continued to have seizures during cognitive decline.

Of the 40 people with epilepsy, 30 (75%) were prescribed ASM monotherapy, 8 (20%) polytherapy, and 2 (5%) individuals declined ASM therapy. Among the 38 people prescribed ASMs, all 30 on monotherapy (100%) and 6 on polytherapy (75%) achieved seizure freedom, which was defined as no seizures for at least 1 year (Kwan et al., 2010). The remaining 2 people trialed multiple ASMs yet continued to have uncontrolled seizures.

### Univariable Analysis of the Characteristics

The univariable comparison of the basic characteristics of people with and without epilepsy is displayed in Table 3. The distribution of education level (*p* = 0.045), subtype of NCD (*p* = 0.034), age at the onset of cognitive decline (*p* = 0.026), and lifestyle factors (smoking and alcohol use, *p* = 0.029 and 0.036, respectively), were significantly different between the two groups. Compared to those without epilepsy, a higher proportion of people with epilepsy achieved tertiary education (47.5 vs. 31.3%) and had younger-onset (age 50 or less) NCDs (42.5 vs. 25.6%). The most common NCD subtypes were unspecified NCD (*n* = 16/40, 40%), AD (*n* = 10/40, 25%), and FTD (*n* = 6/40, 15%) in those with epilepsy, while AD (*n* = 104/387, 26.9%), FTD (*n* = 98/387, 25.3%), and unspecified NCD (*n* = 73/387, 18.9%) were more common in people without epilepsy. Moreover, people with epilepsy had a higher proportion of current smoking status (32.5 vs. 16.8%) and alcohol use (40 vs. 24%) compared to those without epilepsy.

**Table 3:** Characteristics between cohort with and without epilepsy.

**Variables**	**With epilepsy (*n* = 40)**	**Without epilepsy (*n* = 387)**	***P*-value**	****x**^**2**^/*t* value**
**Demographics**				
Female sex, No./total No. (%)	16/40 (40.0)	178/387 (46.0)	0.508	0.526
Age, median (range)	59 (38–85)	59 (33–86)	0.783	n/a^#^
Education, No./total No. (%)			0.045	6.105
No more than basic	10/40 (25.0)	80/379 (20.7)		
High school	11/40 (27.5)	178/379 (46.0)		
Tertiary	19/40 (47.5)	121/379 (31.3)		
Married, No./total No. (%)	26/40 (65.0)	259/383 (67.6)	0.860	0.113
**NCD characteristics**				
Mild NCD, No./total No. (%)	9/40 (22.5)	58/387 (15.0)	0.251	1.547
Dementia subtypes, No./total No. (%)			0.034	10.185
AD	10/40 (25.0)	104/387 (26.9)		
VD	5/40 (12.5)	46/387 (11.9)		
MDD	3/40 (7.5)	66/387 (17.1)		
DLB	2/40(5.0)	22/387 (5.7)		
PDD	0/40 (0)	33/387 (8.5)		
MSA	0/40 (0)	3/387 (0.8)		
PSP	1/40 (2.5)	3/387 (0.8)		
CBD	0/40 (0)	5/387 (1.3)		
FTD	6/40 (15.0)	98/387 (25.3)		
Unspecified ^a^	16/40 (40.0)	73/387 (18.9)		
**NCD onset age, No./total No. (%)**				
Young-onset (<65)	29/40 (72.5)	292/387 (75.5)	0.702	0.169
≤50	17/40 (42.5)	99/387 (25.6)	0.026	5.245
NCD duration (years), median (range)	3.00 (0.5–10)	2.50 (0.5–15)	0.107	n/a^#^
Total NUCOG^b^, mean ± SD	64.89 ± 22.03	66.35 ± 17.13	0.667	0.431
Family history of NCD, No./total No. (%)	13/40 (32.5)	82/387 (21.2)	0.111	2.681
**Neuroimaging**^**c**^**, No./total No. (%)**				
Abnormality	35/40 (87.5)	328/382 (85.9)	0.820	0.081
Cortical atrophy	26/40 (65.0)	233/382 (61.0)	0.733	0.245
Non-specific abnormality	22/40 (55.0)	204/382 (53.4)	0.847	0.037
Small-vessel ischemic change	18/40 (45.0)	152/382 (39.8)	0.612	0.408
SPECT abnormality^d^, No./total No. (%)	26/29 (89.7)	307/339 (90.6)	0.748	0.025
On ADM^e^, No./total No. (%)	6/40 (15.0)	56/387 (14.5)	~1.000	0.008
**Medical history**				
Psychiatric history, No./total No. (%)			0.538	1.354
Depression/anxiety	18/40 (45.0)	145/387 (37.5)		
Other psychiatric disorders	8/40 (20.0)	70/387 (18.1)		
Hypertension, No./total No. (%)	13/40 (32.5)	112/387 (28.9)	0.715	0.222
Diabetes, No./total No. (%)	4/40 (10.0)	58/387 (15.0)	0.487	0.726
Hypercholesterolaemia, No./total No. (%)	14/40 (35.0)	158/387 (40.8)	0.504	0.512
Obstructive sleep apnea, No./total No. (%)	2/40 (5.0)	22/387(5.7)	~1.000	0.032
**Lifestyle factors**				
Current smoking, No./total No. (%)	13/40 (32.5)	65/387 (16.8)	0.029	5.988
Alcohol use, No./total No. (%)	16/40 (40.0)	93/387 (24.0)	0.036	4.863

*AD, Alzheimer disease; VD, Vascular dementia; MD, Movement disorder associated dementia; DLB, Dementia with Lewy bodies; PDD, Dementia associated with Parkinson disease; MSA, Multiple system atrophy; PSP, progressive supranuclear palsy; CBD, corticobasal degeneration; FTD, frontotemporal dementia; ADM, anti-dementia medications*.

# *not available for Mann-Whiteney U-test*.

a *Include 4 people with mixed NCDs (3 AD/VD and 1 FTD/VD), 5 with mild cognitive impairment who did not meet diagnostic criteria for differential diagnoses, and 7 who did not meet the criterion for a specific type in the cohort with epilepsy, and 20 people with mixed NCDs (15 AD/VD, 2 FTD/VD, 2 PDD/VD, and 1 DLB/VD), 9 with mild cognitive impairment who did not meet diagnostic criteria for differential diagnoses, and 44 who did not meet the criterion for a specific type in the cohort without epilepsy*.

b *based on the data of 30 people with epilepsy and 294 people without epilepsy*.

c *Mostly based on MRI, 10 of them based on CT as the people (including 2 people with epilepsy) had only CT but no MRI, 5 people (all without epilepsy) had neither MRI nor CT*.

d *based on the data of 29 people with epilepsy and 339 people without epilepsy*.

e *before onset of epilepsy or before the last follow-up record*.

Other factors including sex, age, family history of dementia, mild or major NCD, duration of cognitive decline prior to data collection time point, extent of cognitive impairment, and neuroimaging findings were not significantly different between people with and without epilepsy.

### Multivariable Analysis for Risk Factors of Having Epilepsy in People With NCDs

The multivariable logistic regression identified NCD subtype, age at onset of cognitive decline, and current smoking status as risk factors for having epilepsy (Table 4). People with FTD and MDD showed significantly lower risk of developing epilepsy compared to unspecified NCD (odds ratio [OR] 0.23, 95% confidence interval [CI] 0.06–0.85, *p* = 0.028 and OR 0.23, 95% CI: 0.08–0.63, *p* = 0.005, respectively), while the risk did not significantly differ for other NCD subtypes. People whose cognitive decline began at age 50 or less had a significantly higher risk of having epilepsy compared to those with cognitive decline starting after age 50 years (OR 2.29, 95% CI: 1.12–4.68, *p* = 0.023). Current smoking status also increased the risk of epilepsy compared to non- or ex-smokers (OR 2.18, 95% CI: 1.04–4.57, *p* = 0.038).

**Table 4:** Multivariable logistic regression in the whole cohort and the young-onset cohort.

	**Whole cohort**			**Young-onset cohort**		
**Variables**	***p***	**OR/β**	**95% CI**	***p***	**OR/β**	**95% CI**
Dementia subtypes	0.026*			0.152		
Unspecified NCD		1/0			1/0	
AD	0.056	0.43/-0.37	0.18–1.02	0.121	0.46/-0.34	0.17–1.23
FTD	0.028*	0.23/-0.64	0.06–0.85	0.012*	0.25/-0.60	0.09–0.74
VD	0.248	0.52/-0.28	0.18–1.57	0.261	0.45/-0.35	0.11–1.82
MDD	0.005*	0.23/-0.64	0.08–0.63	0.998	–	–
Onset age of ≤50	0.023*	2.29/0.36	1.12–4.68	0.024*	2.59/0.41	1.13–5.91
Current smoking	0.038*	2.18/0.34	1.04–4.57	0.006*	3.23/0.51	1.39–7.47
Constant	0.000	0.14/0.85		0.000	0.11/-0.96	

*AD, Alzheimer disease; FTD, frontotemporal dementia; VD, Vascular dementia; MDD, Movement disorder associated dementia; OR, Odds ratio; CI, confidence interval*.

### Sub-analysis of Predictors of Epilepsy Within the Subgroup With Young-Onset NCDs

To test if the results of the whole cohort were applicable to those with young-onset NCDs, we performed a sub-analysis of predictors in this unique subgroup. Dementia subtypes (*p* = 0.02), age at onset of cognitive decline (*p* = 0.01), current smoking status (*p* = 0.008), and current alcohol use (*p* = 0.015) were significantly different between the subgroups with and without epilepsy (Table 5). These factors were then included in a logistic regression model, along with other variables with *p* < 0.2 in the univariable analysis (i.e., age, level of education, duration of cognitive decline, and diabetes). Onset age of cognitive decline, current smoking status, and dementia etiology were selected into the regression model by using forward stepwise method (Table 4). Onset age of cognitive decline ≤50 (OR 2.59, 95% CI: 1.13–5.91, *p* = 0.024) and current smoking status (OR 3.23, 95% CI: 1.39–7.47, *p* = 0.006) were independently associated with increased risk of developing epilepsy. NCD subtype overall was not identified as an independent risk factor for developing epilepsy in those with younger-onset NCDs (*p* = 0.152), although FTD was associated with a lower risk of developing epilepsy compared to unspecified NCD (OR 0.25, 95% CI: 0.09–0.74, *p* = 0.012).

**Table 5:** Characteristics between young-onset NCD people with and without epilepsy.

**Variables**	**With epilepsy (*n* = 29)**	**Without epilepsy (*n* = 292)**	***P*-value**	****x**^**2**^/*t* value**
**Demographics**				
Female sex, No./total No. (%)	12/29 (41.4)	141/292 (48.3)	0.561	0.505
Age, median (range)	53 (38–76)	57 (33–72)	0.130	n/a^#^
Education, No./total No. (%)			0.083	4.803
No more than basic	6/29 (20.7)	47/285 (16.5)		
High school	9/29 (31.0)	147/285 (51.6)		
Tertiary	14 (48.3)	91/285 (31.9)		
Married, No./total No. (%)	17/40 (58.6)	182/289 (63.2)	0.688	0.236
**NCD characteristics**				
Mild NCD, No./total No. (%)	7/29 (24.1)	48/292 (16.4)	0.303	1.101
Dementia subtypes, No./total No. (%)			0.024	10.870
AD	8/29 (27.6)	80/292 (27.4)		
VD	3/29 (10.3)	29/292 (9.9)		
MDD	0/29 (0)	39/292 (13.4)		
DLB	0/29 (0)	9/292 (3.1)		
PDD	0/29 (0)	21/292 (7.2)		
MSA	0/29 (0)	3/292 (1.0)		
PSP	0/29 (0)	2/292 (0.7)		
CBD	0/29 (0)	4/292 (1.4)		
FTD	6/29 (20.7)	89/292 (30.5)		
Unspecified^a^	12/29 (41.4)	55/292 (18.8)		
NCD onset age ≤50, No./total No. (%)	17/29 (58.6)	99/292 (33.9)	0.010	6.983
NCD duration (years), median (range)	3.00 (0.5–10)	3.00 (0.5–15)	0.193	n/a^#^
Total NUCOG^b^, mean ± SD	63.83 ± 20.19	67.20 ± 16.31	0.435	0.793
Family history of NCD, No./total No. (%)	10/29 (34.5)	74/292 (25.3)	0.375	1.141
**Neuroimaging**^**c**^**, No./total No. (%)**				
Abnormality	25/29 (86.2)	244/289 (84.4)	~1.000	0.064
Cortical atrophy	21/29 (72.4)	182/289 (63.0)	0.418	1.017
Non-specific abnormality	13/29 (44.8)	139/289 (48.1)	0.846	0.113
Small-vessel ischemic change	10/29 (34.5)	104/289 (36.0)	~1.000	0.026
SPECT abnormality^d^, No./total No. (%)	20/22 (91.0)	237/263 (90.1)	~1.00	0.014
On ADM^e^, No./total No. (%)	4/29 (13.8)	41/292 (14.0)	~1.00	0.001
**Medical history**				
Psychiatric history, No./total No. (%)	20/29 (69.0)	180/292 (61.6)	0.548	0.602
Depression/anxiety	12/29 (41.4)	112/292 (38.4)		
Other psychiatric disorders	8/29 (27.6)	59/292 (20.2)		
Hypertension, No./total No. (%)	6/29 (20.7)	72/292 (24.7)	0.662	0.226
Diabetes, No./total No. (%)	1/29 (3.4)	42/292 (14.4)	0.149	2.719
Hypercholesterolaemia, No./total No. (%)	12/29 (41.4)	119/292 (40.8)	~1.000	0.004
Obstructive sleep apnea, No./total No. (%)	2/29 (6.9)	14/292 (4.8)	0.646	0.246
**Lifestyle factors**				
Current smoking, No./total No. (%)	12/29 (41.4)	55/292 (18.8)	0.008	8.118
Alcohol use, No./total No. (%)	14/29 (48.3)	74/292 (25.3)	0.015	6.972

*AD, Alzheimer disease; VD, Vascular dementia; MD, Movement disorder associated dementia; DLB, Dementia with Lewy bodies; PDD, Dementia associated with Parkinson disease; MSA, Multiple system atrophy; PSP, progressive supranuclear palsy; CBD, corticobasal degeneration; FTD, frontotemporal dementia; ADM, anti-dementia medications*.

# *not available for Mann-Whiteney U-test*.

a *Include 2 people with mixed NCDs (1 AD/VD and 1 FTD/VD), 4 with mild cognitive impairment who did not meet diagnostic criteria for differential diagnoses, and 6 who did not meet the criterion for a specific type in the cohort with epilepsy, and 13 people with mixed NCDs (10 AD/VD, 2 FTD/VD, 1 PDD/VD, and 1 DLB/VD), 11 with mild cognitive impairment who did not meet diagnostic criteria for differential diagnoses, and 29 who did not meet the criterion for a specific type in the cohort without epilepsy*.

b *based on the data of 24 people with epilepsy and 233 people without epilepsy*.

c *Mostly based on MRI, 6 of them based on CT as the people (all without epilepsy) had only CT but no MRI, 3 people (all without epilepsy) had neither MRI nor CT*.

d *based on the data of 22 people with epilepsy and 263 people without epilepsy*.

e *before onset of epilepsy or before the last follow-up record*.

## Discussion

This study revealed that younger age (≤50 years), NCD of unspecified subtype, and current smoking status were independently associated with development of epilepsy in people with NCDs. We believe these results offer important insights into the intersection of these two common conditions, as the cohort was younger and had a wider range of underlying NCD etiologies compared to previous studies.

Previous studies have also identified that younger age at onset of cognitive decline or dementia is an important risk factor, and sometimes the sole predictor, of dementia-related epilepsy or seizures (Amatniek et al., 2006; Scarmeas et al., 2009; Vossel et al., 2013; Beagle et al., 2017; Zelano et al., 2020). The incidence of unprovoked seizures or new-diagnosis epilepsy for those with NCDs aged 50–59 is almost three times the incidence for those aged 60–69, and eight times the incidence for those aged 70 years or more (Amatniek et al., 2006). The higher risk of epilepsy in younger-onset NCD may be due to epileptic seizures hastening the manifestation of cognitive impairment (Volicer et al., 1995; Lott et al., 2012). In addition, shared common pathophysiological mechanisms, such as unique expression of genes that regulate neural network activity, may underlie these findings (Shea et al., 2016). Supporting this, we found a higher proportion of younger-onset NCD (<50 years) (*n* = 32/84, 38.1%) compared to those >50 years (63/248, 25.4%) had a family history of NCD, indicating a potential hereditary component.

The wide range of etiologies underlying NCDs are associated with different risks for development of epilepsy; our study identified that non-specific-, AD-, and VD-NCD subtypes placed people at highest risk of new-onset epilepsy. Few studies have evaluated the varying association between NCD etiology and occurrence of epileptic seizures, with most previous research focused almost exclusively on people with AD, the commonest NCD (Hesdorffer et al., 1996; Friedman et al., 2012; Imfeld et al., 2013; Vossel et al., 2017). A recent registry-based study found that compared to non-dementia controls, the risk of epilepsy was greatest in those with early-onset (<65 years) AD (HR = 5.83), compared to other NCD subtypes (HR 1.92 for late-onset AD, 2.62 for VD, 3.11 for DLB, 3.79 for PDD, 2.75 for FTD, 2.16 for mixed dementia) (Zelano et al., 2020). This may be due to the younger age of onset, rather than other factors, in the early-onset AD group. However, no subgroup analysis was reported that compared risk of epilepsy in early- vs. late-onset dementia. Previous studies report prevalence of new-onset epilepsy between 1.82 and 6.9% for people with AD (Friedman et al., 2012) and incidence 6.3% for VD (Zelano et al., 2020). The prevalence in our study was higher, likely due to inclusion of more participants with younger age of onset, and inclusion of people whose epilepsy developed up to 10 years before the onset of cognitive impairment. The relatively higher risk of epilepsy for AD- and VD-NCD subtypes may be due to the potential epileptogenic effect of amyloid β and tau pathology, a shared genotype such as APOE4, and increased markers of cerebrovascular disease on brain imaging in people with epilepsy (Sen et al., 2018).

In our study, there was also an association between new-onset epilepsy and unspecified NCD. The higher incidence might due to the add-on effect of mixed etiologies. Further, for the majority of this group, seizures occurred prior to clinically appreciably cognitive decline. This early presentation of seizures may be a manifestation of an underlying pathophysiological mechanism that also contributes to neurocognitive impairment, as opposed to the seizures occurring as a consequence of an advancing neurodegenerative process. In addition, we noted that the incidence of epilepsy was lowest in the subgroup with MDD, especially those with PDD (none of the individuals with PDD had epilepsy). Although some studies have reported a prevalence of epilepsy of 2.6% (Feddersen et al., 2014) and 1.7 increased odds of developing epilepsy (Gruntz et al., 2018) in people with PD, a negative relationship between PD and seizures and seizure-modulating effects of dopamine receptor agonists have also been found (Quesney et al., 1980; Vercueil, 2000), in keeping with our findings.

While seizure onset occurred in close temporal association with NCD onset in our study, the temporal relationship varies widely across the literature. Earlier studies reported that seizures tended to occur in the late stages of dementia (Hauser et al., 1986; Romanelli et al., 1990). In contrast, the majority of recent, large studies found that seizure onset clustered prior to and shortly following a NCD diagnosis (Vossel et al., 2013; Beagle et al., 2017; Zelano et al., 2020). We included people with mild NCDs and so included those in the pre- and early stages of dementia. This “pre- and early” dementia group may be at increased risk of seizures due to the increased cholinergic tone that occurs before the degeneration of cholinergic pathways that is seen in later stages of NCDs (Kam et al., 2016). Studies that report that seizure-onset is associated with later-stage dementia may potentially do so due to a reporting bias, with underreporting and under-recognition of “subtle” non-convulsive seizure types that may occur earlier in the NCD-process (Romanelli et al., 1990). Consistent with some previous studies (Bernardi et al., 2010; Horváth et al., 2018), focal impaired awareness seizures with or without evolution to bilateral tonic clonic seizures, and bilateral tonic clonic seizures, were the most commonly noted seizure type in our NCD cohort. However, some studies reported people with NCDs to have a higher proportion of non-convulsive seizures than convulsive seizure types (Vossel et al., 2013; Beagle et al., 2017). Again, this may reflect a reporting bias favoring recognition of convulsive seizure over non-convulsive seizures. Non-convulsive seizures may be challenging to identify, particularly for individuals with cognitive impairment. Clinicians should be mindful of seizures that manifest with subtle, non-motor semiology, and actively enquire about brief, stereotyped episodes of blank staring, oral or manual automatisms, or other features that may be consistent with focal seizures. As seen in our and previous studies (Belcastro et al., 2007; Vossel et al., 2013), epilepsy was well-controlled with ASMs in most people. Therefore, a high index of clinical suspicion for seizures may facilitate earlier diagnosis and treatment, and this might lead to better prognosis, and otherwise avoidable seizure-related injuries and hospitalizations.

This study reinforced active smoking status as an important predictor of development of epilepsy in NCD cohorts. Smoking has consistently been reported to increase the risk of AD, VD, and all-cause dementia (Anstey et al., 2007; Zhong et al., 2015). Smoking is also a risk factor for seizures. Smokers with epilepsy were found to experience more seizures compared with non-smokers with epilepsy (Johnson et al., 2019), smoking was found to significantly increase the risk of seizures in young adult women aged 25–42 (Dworetzky et al., 2010), and was significantly associated with late-onset epilepsy starting at 60 years or older (Johnson et al., 2018). Our results are in keeping with these studies. As well as being a well–recognized vascular risk factor, one potential hypothesis for the increased risk of epileptic seizures associated with smoking is that it contributes to the atherosclerosis of cerebral vessels and subsequent impairment of neurons, which accelerates the dysfunction of neuro-electrical networks and this leads to the outbreak of epileptic seizures (Sen et al., 2018). Interestingly, in our study, small-vessel ischemic change in neuroimaging was not significantly varied between the subgroups with and without epilepsy. This is consistent with a previous study that showed smoking was associated with late-onset epilepsy even after censoring individuals with stroke (Johnson et al., 2018) and implies possible further pathobiological mechanisms that act separately from the well-described effect on vessels.

Our study reveals several important directions for future research. These include investigating the mechanisms underlying the increased risk of epilepsy for people with younger-onset NCDs vs. older-onset NCDs through techniques such as genetic screening; to confirm the association between various NCD etiology/subtypes and risk of epilepsy; and to elicit the underlying shared mechanisms. Larger numbers of people with specific MDDs and other mixed dementias are needed to better determine how these rarer younger-onset NCDs may be associated with epilepsy risk. As smoking was found to be associated with increased risk of epilepsy and this association seems independent of its effect on cerebral small vessel disease, future studies may focus on identifying additional pathobiological mechanisms above and beyond vascular disease. Finally, tobacco smoking represents a modifiable risk factor, and interventions to reduce or cease smoking should be further investigated as a potential target for reducing risk of new-onset epilepsy in those with NCDs.

Our study has several limitations. Firstly, common to most epidemiology studies in the “real-world setting,” seizure occurrence was primarily based on self-reporting by patients or caregivers. Given that focal seizure semiology is often subtle and some symptoms may overlap with cognitive features of NCDs, the true prevalence of epilepsy may be higher than estimated. Second, information on the exact amount of smoking was not collected as part of routine care, limiting further analysis regarding the possibility of a dose-dependent relationship. Lastly, the study cohort was derived from a tertiary neuropsychiatry service with expertise in young-onset NCDs and so the study cohort may be younger with more severe disease, and so may not be representative of all people with MCI/dementia. Results may therefore not be generalizable to older people with more typical disease.

## Conclusions

A substantial proportion of people with NCDs will develop epilepsy. Clinicians should be mindful that seizures are common in the years just prior to and shortly following an NCD diagnosis and focal to bilateral tonic clonic seizures are the type most likely to reach medical attention. NCD of unclear etiology, younger age at onset of cognitive decline (50 years or less), and current smoking status were independently associated with development of epilepsy. Early identification of seizures is important, particularly as ASM monotherapy is effective in the majority of cases.

## Data Availability Statement

The raw data supporting the conclusions of this article will be made available by the authors, without undue reservation.

## Ethics Statement

The studies involving human participants were reviewed and approved by The RMH Human Research Ethics Committee. Written informed consent for participation was not required for this study in accordance with the national legislation and the institutional requirements.

## Author Contributions

XW, DV, and PK designed the study. XW, SL, and EF collected the data. XW performed the statistical analysis with assistance from ZC. XW drafted the manuscript, which was critically revised by SL, EF, DV, and PK. All authors approved the final version of the manuscript.

## Conflict of Interest

The authors declare that the research was conducted in the absence of any commercial or financial relationships that could be construed as a potential conflict of interest.
